# Side-Opened Hollow Fiber-Based SPR Sensor for High Refractive Index Detection

**DOI:** 10.3390/s24134335

**Published:** 2024-07-04

**Authors:** Ge Meng, Nannan Luan, Hao He, Fan Lei, Jianfei Liu

**Affiliations:** 1 School of Electronics and Information Engineering, Hebei University of Technology, Tianjin 300401, China; 202221902017@stu.hebut.edu.cn (G.M.); hehao@hebut.edu.cn (H.H.); jfliu@hebut.edu.cn (J.L.); 2 Key Laboratory of Natural Resources Monitoring and Supervision in Southern Hilly Region, Ministry of Natural Resources, Changsha 410000, China; xyp@img.net

**Keywords:** optical fiber sensors, optical sensors, optical fibers, refractive index, surface plasmon resonance

## Abstract

To facilitate the sensor fabrication and sensing operation in microstructured optical fiber-based surface plasmon resonance (SPR) sensors for high refractive index (RI) detection, we propose a special hollow fiber-based SPR sensor that comprises an opening on its body side and a thin gold layer coated on its outer surface. The analyte is able to flow into the hollow core through the side-opening to form new fiber core, with the Gaussian-like mode propagating in it. We investigate the sensing performance of the proposed sensor in a higher RI range of 1.48 to 1.54 at two feasible schemes: one is to only fill the fiber core with analyte (Scheme A), and the other is to directly immerse the sensor in the analyte (Scheme B). The results demonstrate that our sensor exhibits higher wavelength sensitivity at Scheme A with a maximum wavelength sensitivity of 12,320 nm/RIU, while a greater amplitude sensitivity was found at Scheme B with a maximum amplitude sensitivity of 1146 RIU^−1^. Our proposed sensor features the advantages of simple fabrication, flexible operation, easy analyte filling and replacing, enhanced real-time detection capabilities, high RI detection, and very high wavelength sensitivity and amplitude sensitivity, which makes it more competitive in SPR sensing applications.

## 1. Introduction

Surface plasmon resonance (SPR) is the resonant excitation of free electron density oscillation at the interface of a metal and a dielectric (it can be solid, liquid, or gas) or any two materials having opposite signs of dielectric permittivity [1,2,3]. As a result, SPR is very sensitive to small variations in the refractive index (RI) of the medium adjacent to the metal, which constitutes the core of most SPR sensors. The earliest SPR sensing devices were based on prisms, such as the well-known Kretschmann–Raether prism, which can achieve high sensitivity, but they have the disadvantages of being bulky in size, limited mechanical reliability, costly manufacturing, and difficulty in remote measurement [4,5]. As an alternative to the Kretschmann–Raether prism configuration, the optical fiber-based SPR sensor, due to its unique advantages such as miniaturization, ease of implementation, fast response, cost-effectiveness, and remote sensing, has gathered continuous research interest in many fields including biosensing [6], environmental monitoring [7], food safety [8], medicinal development [9], et al.

In most fiber-based SPR sensors, the fiber core serves as a prism, the fiber cladding is partially or completely removed and coated with a metal layer, and eventually surrounded by analyte [1,2,3]. Once the phase matching condition is satisfied, theoretically, that is the equality of propagation constants (or effective refractive indexes) between a plasmonic mode and a core-guided mode at a specific wavelength, the energy of the core-guided mode is transferred to the plasmonic mode, and results in a dip or a peak in SPR spectra [1,2]. As the plasmonic mode is highly sensitive to the RI of the analyte neighboring metal layer, the variations in analyte RI could change the SPR spectra, and hence, can be detected by monitoring the SPR spectra changes. In addition, the effective refractive index (*n*_eff_) of the core-guided mode closely approximates the RI of the core materials, that is typically 1.45 for the silica. The *n*_eff_ of the plasmonic mode is typically close to that of the surrounding analytes, which are 1.0 for the air and 1.33 for the water [10,11,12]. Only at higher frequencies does the *n*_eff_ of the plasmonic mode become sufficiently high to be equal to that of the core-guided mode. However, at high frequency, the depth of plasmon penetration into the analyte is limited, which could reduce the sensitivity of the sensor [10,11,12,13]. On the other hand, for the satisfactory requirement of total internal reflection (TIR) in fiber sensing, the RI of the fiber core should be higher than that of the cladding. Therefore, most fiber-based SPR sensors can only detect analytes with RI lower than that of the fiber material [1,2,3,4,13,14]. For the analytes with RI higher than that of the fiber material, such as organic chemical liquid analytes containing benzene, toluene [15], nitrobenzene, phenylamine [16], et al., it is difficult to deal with.

In order to address the above issues, recently, the microstructured optical fiber (MOF) has been proposed to be applied in the SPR sensors because they effectively facilitate the phase matching issue and also achieve the detection of high RI by specific design [11,12,17,18,19,20,21,22,23,24,25,26]. Most of the MOF-based SPR sensors for high RI detection are based on multi-core structures where discrete core-guided modes interact with each other to form a core-guide super-mode [17,18,19,20,21]. However, these designs pose difficulties with the selective excitation of the core-guide super-mode, thus complicating the operation of these sensors. Moreover, the majority of these sensors require the analytes to be filled into closed air holes or narrow grooves, and this process results in a slower analyte filling speed, which consequently increase the response time of the sensor and restrict their real-time sensing capabilities [17,18,20]. Hollow fiber (HF)-based SPR sensors, which are composed of a supporting tube with a metal layer coated on its inner surface and liquid analytes filled in the tube, are one of the simplest structures of MOF-based SPR sensors for high RI detection and are thus receiving increasing attention [22,23,24,25,26]. Unlike most fiber-based SPR sensors, the HF-based SPR sensor holds the liquid analytes inside the hollow core and light can propagate in the analyte. Due to its requirement for TIR, the liquid analyte should have a RI higher than that of the fiber material. Consequently, the HF-based SPR sensor has a natural advantage in the detection of analytes with high RI. So far, studies of utilizing such kinds of HF-based SPR sensors to detect liquids with high RI have been reported both theoretically and experimentally [22,23,24,25,26]. However, it is noteworthy that the bore size of the hollow core in these sensors is as small as several microns and the deposition of the thin metal layer on the inner wall of the hollow core, emptying, and re-filling the analyte solution are very complicated processes [22,23,24,25,26]. As a result, it is almost impossible to achieve real-time detection and fast responses by employing these HF-based SPR sensors.

To break through the above issues, in this paper, we propose a side-opened HF-based SPR sensor for high RI detection, where its outer surface is coated with a thin gold layer and its structure is based on a single core which uses a Gaussian-like mode as the core-guided mode. Such a design eases the difficulties in sensor fabrication and sensing operation. The introduction of side-opening is convenient for filling and replacing analytes, and also quickening the filling speed of analytes to enhance the ability of real-time detection. We investigate the sensing performance of the proposed sensor for high RI detection at two operation schemes, that are only filling the hollow core of the sensor with analyte and immersing the sensor in analyte, and also discuss the effect of opening width on the sensing performance of the sensor.

## 2. Sensor Configuration and Theoretical Model

**Figure 1:**
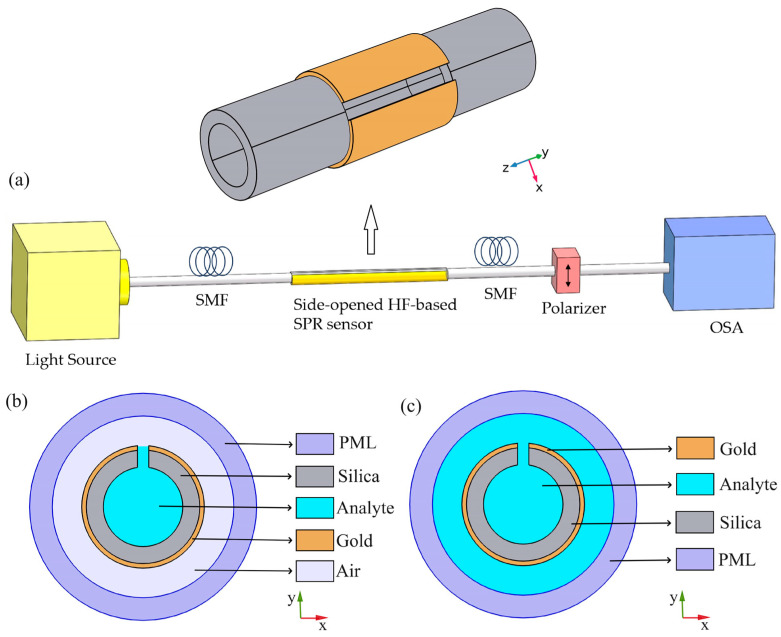
Schematics of the side-opened HF-based SPR sensor. (**a**) 3D-view and the experimental setup of the sensor for high RI detection. (**b**) Cross-section view of Scheme A which is to only fill the hollow core with analyte. (**c**) Cross-section view of Scheme B which is to immerse the sensor in analyte.

The 3D schematic diagram of the side-opened HF-based SPR sensor is shown in Figure 1a, where the supporting tube is a fused silica capillary with an inner diameter of 8 μm and a thickness of 1 μm. An opening with a width of 1 μm is fabricated on the side of the HF by using proven technologies such as focused ion beam [27] or femtosecond laser techniques [28], which can accelerate the liquid analyte filling speed [29,30]. In addition, a gold layer with a thickness of 40 nm is deposited on the outer surface of the supporting tube, which is much easier to be implemented than that deposited on the inner wall of hollow core [17,18,20]. And deposition of the gold layer can be performed using wet chemistry deposition or chemical vapor deposition (CVD) [31,32]. Figure 1a also shows the experimental setup where a wide-band light source is used to launch into single-mode fibers (SMFs) and couple to the side-opened HF-based SPR sensor. A polarizer is required in this system to select the *x*-polarized mode. Finally, the transmitted light is coupled to the optical spectrum analyzer (OSA) for analysis. According to the structural characteristics of sensors, for the sensing operation, there are two sensing schemes in this design, one is to only fill the hollow core of the sensor with the liquid analyte (Scheme A) as shown in Figure 1b, and the other is to directly immerse the sensor into the liquid analyte (Scheme B) as illustrated in Figure 1c.

In order to investigate the performance of the proposed sensor at the two schemes for sensing. We use the finite element method (FEM) to simulate the characteristics of the propagation modes. The circular perfectly matched layer (PML) is placed outside the HF to absorb the reflected light at the boundary layer, as shown in Figure 1b,c, which is employed to advance the accurate calculation of propagation modes in optical fibers [18]. In the simulation model, the fiber material is silica and its RI (*n*(*λ*)) is a function of the wavelength (*λ*), which can be calculated on the basis of the Sellmeier dispersion relation as follows [33]:

(1) $$n^{2}(\lambda )=1+\frac{A_{1}\lambda^{2}}{\lambda^{2}-B_{1}}+\frac{A_{2}\lambda^{2}}{\lambda^{2}-B_{2}}+\frac{A_{3}\lambda^{2}}{\lambda^{2}-B_{3}}$$

where A_1_–A_3_ and B_1_–B_3_ are the Sellmeier coefficients. In this case, the values of the coefficients are 0.69616300, 0.407942600, and 0.89779400, and 4.67914826 × 10^−3^ μm^2^, 1.35120631 × 10^−2^ μm^2^, and 97.9340025 μm^2^, respectively. The dielectric constant of gold (ε_Au_) can be expressed by the Drude–Lorentz model [34]:

(2) $$\varepsilon_{\mathrm{Au}}=\varepsilon_{\infty }-\frac{\omega_{D}^{2}}{\omega (\omega +j\gamma_{D})}-\frac{\Delta \varepsilon \Omega_{L}^{2}}{(\omega^{2}-\Omega_{L}^{2})+j\Gamma_{L}\omega }$$

where *ε*_∞_ = 5.9673 is the permittivity at infinite frequency, Δ*ε* = 1.09 is a weighting factor, *ω* is the angular frequency which can be calculated by *ω* = 2πc/*λ*, and c is the velocity of light in vacuum. Moreover, *ω*_D_ and *γ*_D_ denote the plasma frequency and damping frequency, respectively, where *ω*_D_/2π = 2113.6 THz and *γ*_D_/2π = 15.92 THz. Γ_L_/2π = 104.86 THz and Ω_L_/2π = 650.07 THz are the spectral width and oscillator strength, respectively, of the Lorentz oscillators.

## 3. Results

### 3.1. Dispersion Relation

**Figure 2:**
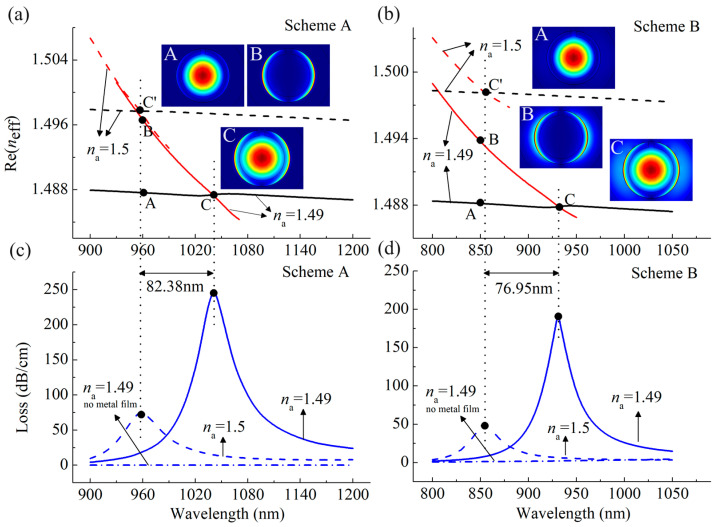
Dispersion relations of the core-guided mode and the plasmonic mode of the proposed sensor at (**a**) Scheme A and (**b**) Scheme B when the *n*_a_ changes from 1.49 to 1.50. Insets show the evolution of electric field distributions of the corresponding modes. Loss spectra of the core-guided mode of the sensor at (**c**) Scheme A and (**d**) Scheme B when the *n*_a_ changes from 1.49 to 1.50. For comparison, the blue dash-dotted lines show the losses of the core-guided mode without gold coating at *n*_a_ = 1.49.

Figure 2a,b depict the dispersion relation between the core-guided mode (black line) and plasmonic mode (red line) when the refractive index of the analyte (*n*_a_) is 1.49 and 1.5 for the case of Schemes A and B, respectively. Here we choose the Gaussian-like mode as the core-guided mode since it is the most suitable for excitation by standard Gaussian laser sources [10], and employ the *x*-polarized core-guided mode to evaluate the sensing performance of the sensor because it can better couple to the *x*-polarized plasmonic mode that has two bright regions distributed on the gold layer surface at two sides of the fiber (see insets in Figure 2a for example), thus providing better sensing performance than the *y*-polarized core-guided mode [35]. Taking *n*_a_ at 1.49 as an example, the insets show the evolution of the electric field distributions when the core-guided mode is coupled to the plasmonic mode. It can be observed that, at non-resonance wavelengths, the mode field of the core-guided mode as seen from insets A of Figure 2a,b is almost distributed in the core region, and that of the plasmonic mode as shown in insets B of the figure predominantly resides on the surface of the gold film. While at resonance wavelengths, the partial energy of the core-guided mode is transferred to the plasmonic mode, as insets C illustrate. At this moment, the dispersion curves of the two modes intersect at the wavelengths of 1040.97 nm and 931.2 nm (phase matching point), respectively, where the real part of the effective refractive index (Re(*n*_eff_)) of the two modes is equal. This process causes a sharp peak at this wavelength range in the loss spectra as shown by the blue line displayed in Figure 2c,d. For comparison, we also present the losses of the core-guided mode in the absence of a metal coating in the blue dash-dotted line. It can be found that the line is a smooth straight line, which means there is no resonance occurring for the case when no gold film is presented. 

Variations in *n*_a_ will result in changes in the dispersion relation between the core-guided mode and the plasmonic mode of the SPR sensor, thereby altering the loss spectra. Accordingly, different *n*_a_ can be identified through the analysis of alterations in the loss spectra. Two distinct detection approaches have been developed based on this phenomenon, namely, the wavelength interrogation approach and the amplitude interrogation approach [12]. In the former, shifts in the resonance wavelengths are employed to characterize the *n*_a_, while in the latter, changes in the transmission loss spectra at a single wavelength are utilized to determine the *n*_a_ [12]. As shown in Figure 2a,b, the variations in *n*_a_ affect the *n*_eff_ curves of the sensor both at Schemes A and B, hence the SPR spectra of the sensor. However, it is worthy of note that in the case of Scheme A, where the analyte is solely infused into the fiber core, the changes in *n*_a_ only affect the *n*_eff_ of the core-guided mode, as shown in Figure 2a, while in the case of Scheme B, where the analyte exists both in the fiber core and the bordering region of the gold film, the alterations of *n*_a_ impact both the *n*_eff_ of the core-guided mode and the plasmonic mode, as seen in Figure 2b. Consequently, the aforementioned difference leads to a discrepancy in the loss spectra between the two schemes, and thus the sensing performance for the RI detection in both schemes.

### 3.2. Wavelength Sensitivity

In the wavelength interrogation approach, changes in *n*_a_ are detected by measuring the displacement of the resonance wavelengths (*λ*), and the wavelength sensitivity can be defined as equation [12]:

(3) $$S_{\lambda }(\mathrm{nm}/\mathrm{RIU})=\frac{\Delta \lambda }{\Delta n_{a}}$$

where Δ*λ* means the shift of the resonance wavelength and Δ*n*_a_ denotes the variation of *n*_a_.

**Figure 3:**
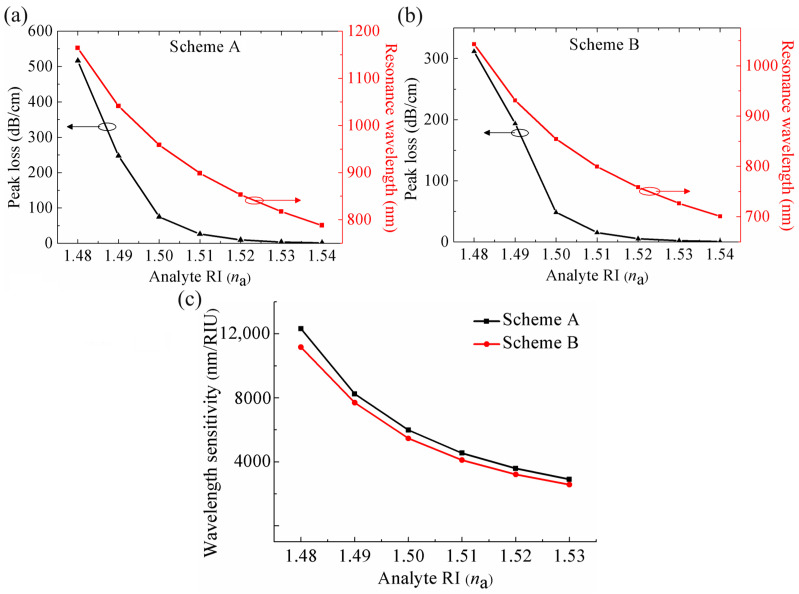
Resonance wavelengths and peak losses of the sensor with different *n*_a_ at (**a**) Scheme A and (**b**) Scheme B. (**c**) Wavelength sensitivities of the sensor with different *n*_a_ at the two schemes.

Figure 3a,b present the variations of peak losses and resonance wavelengths under different *n*_a_ at the two schemes, respectively. It can be found that, in contrast to the MOF-based SPR sensors for low RI detection where the resonance wavelength shifts towards a longer wavelength as *n*_a_ increases [11,12], the resonance wavelength of the proposed SPR sensor for high RI detection shifts towards the shorter wavelength with an increase in *n*_a_. This is attributed to the position of the analyte filling. In the case of low RI detection, the analyte is basically filled in the air holes coated with a metal layer in the fiber cladding and the changes in *n*_a_ mainly affect the *n*_eff_ of the plasmonic mode, thus the phase matching point is located at a longer wavelength with increasing *n*_a_ [11,12]. While in our sensor for high RI detection, the analyte is primarily filled in the fiber core and the variation of *n*_a_ predominantly changes the *n*_eff_ of the core-guided mode, thus the phase matching point shifts towards a shorter wavelength as *n*_a_ increases. 

In addition, it also can be observed that the displacement of the resonance wavelength caused by the two adjacent *n*_a_ variations at Scheme A is longer than that at Scheme B. This is due to the fact that at Scheme A, the increase in *n*_a_ only causes an increase in the *n*_eff_ of the core-guided mode; the upward shift of the core-guided mode dispersion curves generates a left shift of the resonance wavelength as the shift of phase matching point C to C’ shown in Figure 2a. While at Scheme B, increasing *n*_a_ not only induces an increase in the *n*_eff_ of the core-guided mode but also the *n*_eff_ of plasmonic mode; the joint upward shift of the two modes’ dispersion curves also leads to a left shift of the resonance wavelength, but the extent of the left shift is not larger than that at Scheme A, which can be seen from the displacement of the phase matching point C to C’ in Figure 2b. This phenomenon results in distinct sensing performance at the two schemes in wavelength interrogation. According to Equation (3), for example, with *n*_a_ changing from 1.48 to 1.49, the sensor at Scheme A obtains a wavelength sensitivity of 12,320 nm/RIU and the sensor at Scheme B achieves a wavelength sensitivity of 11,165 nm/RIU. The wavelength sensitivity of the sensor with different *n*_a_ at the two schemes are shown in Figure 3c, from which it can be seen that the wavelength sensitivity gradually decreases with an increase in *n*_a_. Note that the wavelength sensitivity at Scheme A under different *n*_a_ is consistently higher than that at Scheme B, which means that in wavelength interrogation, the sensor at Scheme A exhibits a superior sensing performance compared to that at Scheme B.

### 3.3. Amplitude Sensitivity

**Figure 4:**
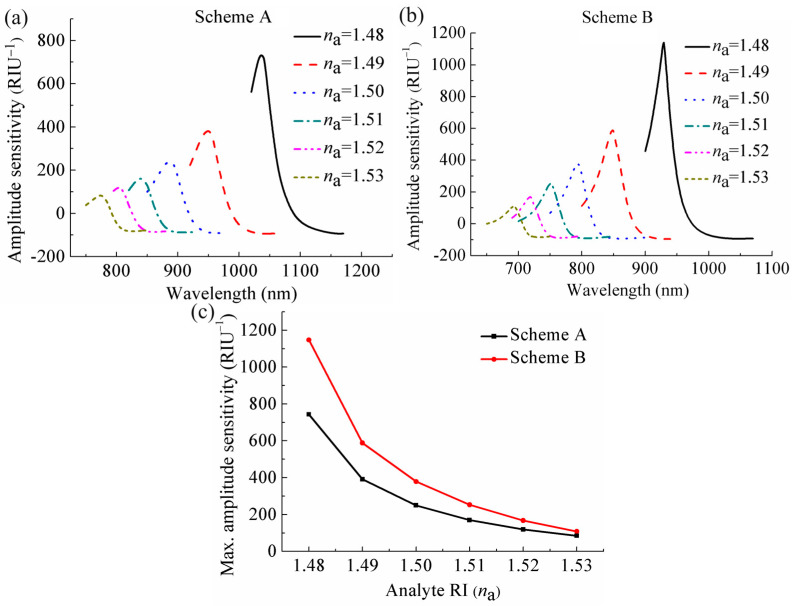
Amplitude sensitivities of the sensor under different *n*_a_ at (**a**) Scheme A and (**b**) Scheme B. (**c**) Comparison of the maximum amplitude sensitivity under different *n*_a_ at the two schemes.

In the amplitude interrogation approach, the variation of *n*_a_ can be detected by comparing the transmission spectra before and after a change in *n*_a_ at a single wavelength. It is cost effective and less complex than the wavelength interrogation method, because it does not require spectral manipulation, and the amplitude sensitivity can be given as follows [12]:

(4) $$S_{A}(\lambda )=-\frac{1}{\alpha (\lambda ,n_{a})}\frac{\partial \alpha (\lambda ,n_{a})}{\partial n_{a}}$$

where α(*λ*, *n*_a_) refers to the total loss at a certain *n*_a_ under a specific wavelength *λ*, and ∂*α*(*λ*,*n*_a_) expresses the variation in loss caused by a change in *n*_a_ (∂*n*_a_). Figure 4a,b illustrate the curves of amplitude sensitivity with wavelength altering under different *n*_a_ at the two schemes. It can be observed that with *n*_a_ changing from 1.48 to 1.49, the sensor at Scheme A reaches a maximum amplitude sensitivity of 743 RIU^−1^ at a wavelength of 1037 nm, while the sensor at Scheme B achieves a maximum amplitude sensitivity of 1146 RIU^−1^ at a wavelength of 930 nm. The data obtained reveal that the maximum amplitude sensitivity at Scheme B is significantly higher than that at Scheme A. The main reason for this is that the overall loss of the sensor *α*(*λ*, *n*_a_) at Scheme B is lower than that at Scheme A under the same *n*_a_, and thus leads to a higher maximum amplitude sensitivity according to Equation (4). Figure 4c presents the maximum amplitude sensitivity curves of the sensor under different *n*_a_ at the two schemes. It can be seen that with an increase of *n*_a_, there is a corresponding reduction in the maximum amplitude sensitivity. Furthermore, the maximum amplitude sensitivities of the sensor at Scheme B under different *n*_a_ are all higher than that at Scheme A, indicating that the sensing performance of the sensor at Scheme B is significantly greater than that at Scheme A.

The above results present the sensing performance of the proposed sensor at the two schemes in two detection approaches: wavelength interrogation approach and amplitude interrogation approach. It can be found that a greater wavelength sensitivity is achieved when the proposed sensor is only filled in the fiber core (Scheme A), while a higher amplitude sensitivity is obtained by immersing the proposed sensor in analyte (Scheme B). One should be aware that the enhancement of amplitude sensitivity is considerably more pronounced than that of wavelength sensitivity. With regard to the *n*_a_ changes from 1.48 to 1.49, the wavelength sensitivity of 12,320 nm/RIU at Scheme A is 1.103 times greater than that at Scheme B of 11,165 nm/RIU, while the maximum amplitude sensitivity of Scheme B at 1146 RIU^−1^ is 1.542 times more than that at Scheme A of 743 RIU^−1^. Moreover, it is also worth noting that the operation process of Scheme B is more straightforward than that of Scheme A, which requires a more precise filling of the analyte only into the hollow core through the side-opening. Table 1 provides a performance comparison of the side-opened HF-based SPR sensor with other MOF-based SPR sensors for high RI detection. It can be seen that in order to achieve detection of high RI, some of the MOF-based SPR sensors need complicated structures (e.g., multi-core structures) and coating a gold layer inside closed holes or narrow grooves [17,18,20,25]. Our sensor not only has a simple structure and outside coating gold layer but also provides very high wavelength sensitivity and amplitude sensitivity. Moreover, compared with the HF-based SPR sensors for high RI detection that require a gold coating layer and filling and replacing analytes in closed holes [24,25], our side-opened HF-based SPR sensor enables easier analyte filling and replacing and also enhanced real-time detection capabilities.

## 4. Discussion

**Figure 5:**
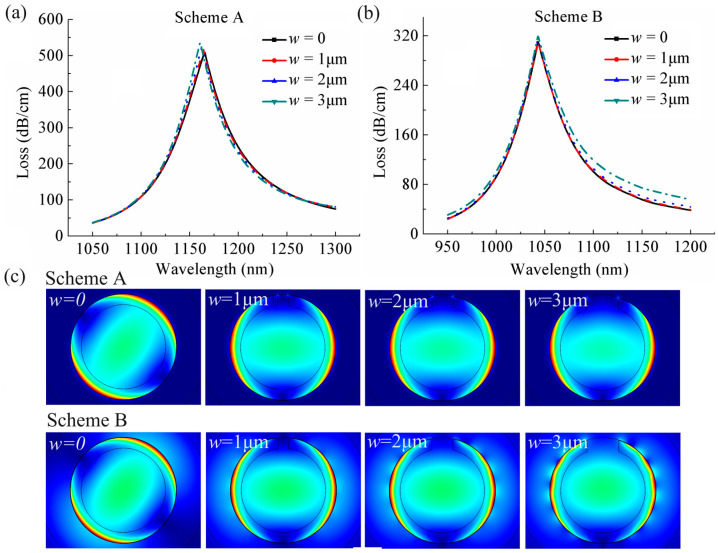
Loss spectra of the core-guided mode of the sensor with *n*_a_ = 1.48 under different *w* at (**a**) Scheme A and (**b**) Scheme B. (**c**) The electric field distribution of the core-guided mode of the sensor with *n*_a_ = 1.48 under different *w* at their respective resonance wavelengths.

**Figure 6:**
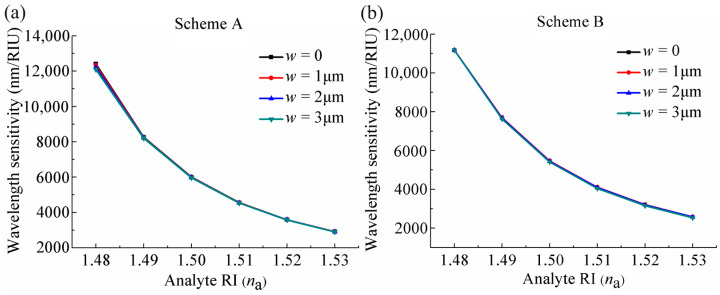
Variations of wavelength sensitivities of the sensor with *n*_a_ changing under different *w* at (**a**) Scheme A and (**b**) Scheme B.

In this sensor, the side opening is designed to allow for easy filling and replacing of analytes, speeding up the liquid filling process to improve the ability of real-time detection, and a wider opening width will be more conducive to promote this process. Therefore, it is necessary to investigate the effect of the opening width on sensing performance. Figure 5a,b present the losses curves of the core-guided mode for opening width *w* of 1 μm, 2 μm, and 3 μm when *n*_a_ is 1.48 at Schemes A and B, respectively. For the purpose of comparison, we also give the loss curves for the case without opening, i.e., *w* = 0. It can be observed that the resonance wavelengths are almost unaffected by the size of the opening, both in the case of Scheme A and of Scheme B. According to Equation (3), the wavelength sensitivity is only related to the resonance wavelength; consequently, it exhibits minimal alteration caused by the *w*, as illustrated in Figure 6a,b, which plot the wavelength sensitivity curves with *n*_a_ altering under different *w* at the two schemes.

**Figure 7:**
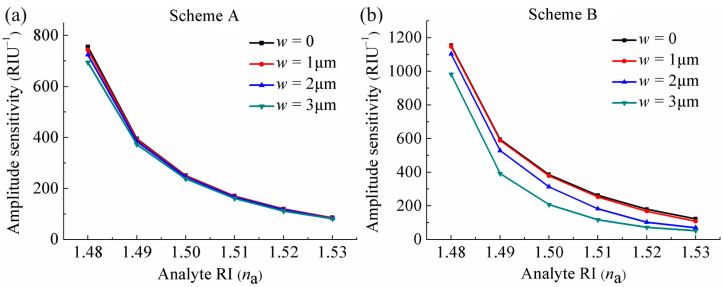
Variations of the maximum amplitude sensitivities of the sensor with *n*_a_ changing under different *w* at (**a**) Scheme A and (**b**) Scheme B.

It can be seen from the Figure 5a,b that the losses increase slightly as the opening becomes wider at the two schemes, which also can be observed by the electric field distribution of the core-guided mode under different *w* when resonance occurs at the two schemes in Figure 5c, where the brightness of the core region decreases as *w* increases. According to Equation (4), the amplitude sensitivity is inversely proportional to the losses of the core-guided mode (*α*(*λ*, *n*_a_)), thus, it is more obviously affected by the wider opening, as shown in Figure 7a,b, which present the maximum amplitude sensitivity curves with changes in *n*_a_ under different *w* at the two schemes. It can be seen that the maximum amplitude sensitivity decreases more significantly with increasing *w*. In addition, it is interesting to note that compared to the amplitude sensitivity of the sensor at Scheme A, Scheme B displays a more pronounced decline in sensitivity with increasing *w*. This can be explained by Figure 5a,b, that as *w* increases, the losses of the core-guided mode of the sensor partially increase at Scheme A, while it has an overall increase at Scheme B, and it is negatively correlated to the amplitude sensitivity, as stated in Equation (4).

Generally, the wider opening facilitates the filling and replacing of analytes, as well as enhances the real-time detection capability due to faster filling speeds. Meanwhile, it has a minimal effect on resonance wavelength and a slight effect on the losses of the core-guided mode, and thus stable wavelength sensitivity and reduced amplitude sensitivity.

## 5. Conclusions

In this paper, we propose a side-opened HF-based SPR sensor for high RI detection, which is coated with a thin gold layer on its outer surface, is infiltrated by analyte through the side-opening, and utilizes the Gaussian-like mode propagation in the analyte core as the core-guided mode. The purpose of this design is not only to reduce the difficulty of the sensor fabrication and sensing operation but also facilitate the filling and replacing of analytes by introducing a side-opening, as well as accelerate the filling speed of analytes to enhance the capability of real-time detection. Based on the characteristics of the sensor structure, we propose two operation schemes, one is to only fill the hollow core of the sensor with analyte (Scheme A) and the other is to immerse the sensor in analyte (Scheme B), and investigate the sensing performance of the sensor for high RI detection at these two schemes. The results indicate that the proposed sensor displays superior wavelength sensitivity at Scheme A, while greater amplitude sensitivity is seen at Scheme B. In addition, the size of the opening has a minimal effect on wavelength sensitivity, while it has an apparent impact on the amplitude sensitivity. Compared to most other MOF-based SPR sensors for high RI detection, the proposed sensor exhibits more competitiveness in sensor fabrication and operation, achieves a greater improvement in the filling and replacing of analytes, enhances the ability of real-time detection, and also has very high wavelength sensitivity and amplitude sensitivity.

## Figures and Tables

**Table 1:** Performance comparison of the side-opened HF-based SPR sensor with other MOF-based SPR sensors.

Fiber Structure	Coating	RI Range	Interrogation Method	Sensitivity	Ref.
Multi-core MOF	Inside closed hole	1.43–1.53	Wavelength	9231 nm/RIU	[17]
			Amplitude	N/A	
Four-core MOF	Inside closed hole	1.42–1.52	Wavelength	12500 nm/RIU	[18]
			Amplitude	549.6 RIU^−1^	
Dual-core D-shaped MOF	Silver nanowire	1.35–1.5	Wavelength	3400 nm/RIU	[19]
			Amplitude	N/A	
H-shaped MOF	Inside narrow grooves	1.33–1.49	Wavelength	25900 nm/RIU	[20]
			Amplitude	N/A	
Dual-core D-shape MOF	External	1.45–1.48	Wavelength	8000 nm/RIU	[21]
			Amplitude	700 RIU^−1^	
Closed HF	Silver nanowire	1.47–1.51	Wavelength	16200 nm/RIU	[24]
			Amplitude	N/A	
Closed HF	Inside closed hole	1.51–1.58	Wavelength	7111 nm/RIU	[25]
			Amplitude	N/A	
Side-opened HF	External	1.48–1.54	Wavelength	12320 nm/RIU	This work
			Amplitude	1146 RIU^−1^	

## Data Availability

Data are contained within the article.
